# Topical Sustained-Release Dexamethasone-Loaded Chitosan Nanoparticles: Assessment of Drug Delivery Efficiency in a Rabbit Model of Endotoxin-Induced Uveitis

**DOI:** 10.3390/pharmaceutics15092273

**Published:** 2023-09-03

**Authors:** Musaed Alkholief, Mohd Abul Kalam, Mohammad Raish, Mushtaq Ahmad Ansari, Nasser B. Alsaleh, Aliyah Almomen, Raisuddin Ali, Aws Alshamsan

**Affiliations:** 1 Department of Pharmaceutics, College of Pharmacy, King Saud University, P.O. Box 2457, Riyadh 11451, Saudi Arabia; makalam@ksu.edu.sa (M.A.K.); mraish@ksu.edu.sa (M.R.); ramohammad@ksu.edu.sa (R.A.); 2 Department of Pharmacology and Toxicology, College of Pharmacy, King Saud University, P.O. Box 2457, Riyadh 11451, Saudi Arabia; muansari@ksu.edu.sa (M.A.A.); nbalsaleh@ksu.edu.sa (N.B.A.); 3 Department of Pharmaceutical Chemistry, College of Pharmacy, King Saud University, P.O. Box 2457, Riyadh 11451, Saudi Arabia; alalmomen@ksu.edu.sa

**Keywords:** dexamethasone, chitosan-nanoparticles, hyaluronic-acid, ocular-pharmacokinetics, endotoxin, uveitis, cytokines, histopathology

## Abstract

Uveitis is an ocular illness that if not treated properly can lead to a total loss of vision. In this study, we evaluated the utility of HA-coated Dexamethasone-sodium-phosphate (DEX)-chitosan nanoparticles (CSNPs) coated with hyaluronic acid (HA) as a sustained ocular delivery vehicle for the treatment of endotoxin-induced-uveitis (EIU) in rabbits. The CSNPs were characterized for particle size, zeta potential, polydispersity, surface morphology, and physicochemical properties. Drug encapsulation, in vitro drug release, and transcorneal permeation were also evaluated. Finally, eye irritation, ocular pharmacokinetics, and pharmacodynamics were in vivo. The CSNPs ranged from 310.4 nm and 379.3 nm pre-(uncoated) and post-lyophilization (with HA-coated), respectively. The zeta potentials were +32 mV (uncoated) and −5 mV (HA-uncoated), while polydispersity was 0.178–0.427. Drug encapsulation and loading in the CSNPs were 73.56% and 6.94% (uncoated) and 71.07% and 5.54% (HA-coated), respectively. The in vitro DEX release over 12 h was 77.1% from the HA-coated and 74.2% from the uncoated NPs. The physicochemical properties of the CSNPs were stable over a 3-month period when stored at 25 °C. Around a 10-fold increased transcorneal-flux and permeability of DEX was found with HA-CSNPs compared to the DEX-aqueous solution (DEX-AqS), and the eye-irritation experiment indicated its ocular safety. After the ocular application of the CSNPs, DEX was detected in the aqueous humor (AH) till 24 h. The area under the concentrations curve (AUC_0–24h_) for DEX from the CSNPs was 1.87-fold (uncoated) and 2.36-fold (HA-coated) higher than DEX-AqS. The half-life (t_1/2_) of DEX from the uncoated and HA-coated NPs was 2.49-and 3.36-fold higher, and the ocular MRT_0-inf_ was 2.47- and 3.15-fold greater, than that of DEX-AqS, respectively. The EIU rabbit model showed increased levels of MPO, TNF-α, and IL-6 in AH. Topical DEX-loaded CSNPs reduced MPO, TNF-α, and IL-6 levels as well as inhibited NF-κB expression. Our findings demonstrate that the DEX-CSNPs platform has improved the delivery properties and, hence, the promising anti-inflammatory effects on EIU in rabbits.

## 1. Introduction

Uveitis is a prevalent condition with a prevalence of 24.9 cases per 100,000 persons that affects either gender population significantly by geographic location and all ages of patients [1]. Among the different types of uveitis, the anterior uveitis is the most prevalent and the posterior uveitis is the least prevalent [2,3]. The rodent model for endotoxin-induced uveitis (EUI) is widely applied to evaluate anterior uveitis [4]. EIU is an acute inflammation of the anterior chamber of the eye that is induced by the intravitreal injection of endotoxins (also known as lipopolysaccharides; LPSs) [4]. Prostaglandin E_2_ (PGE_2_) and tumor necrosis factor-alpha (TNF-α) are the primary meditates associated with EIU [5,6], but other chemokines and cytokines have also been shown to play a role in EIU [6,7]. Thus, suppressing pro-inflammatory mediators is a key strategy in controlling and resolving EIU.

Previous studies have revealed that systemic glucocorticoids such as dexamethasone (DEX) (9α-fluoro-16α-methyl-11β,17α,21-trihydroxy-1,4-pregnadiene-3,20-dione) mitigate the production of inflammatory mediators in EIU animal models [5,8]. DEX is a long-acting synthetic glucocorticoid and, among other corticosteroids, DEX sodium phosphate has the highest potency and efficacy against ocular inflammatory conditions [9,10]. It acts by binding to corticosteroid receptors found in human trabecular meshwork cells and the iris as well as the ciliary bodies of rabbit eyes. DEX sodium phosphate reduces pain and swelling by inhibiting the phospholipase-A_2_ pathway and the associated inflammatory eicosanoids, including prostaglandins and leukotrienes. Thus, it is often used to reduce injury-, surgery-, and infection-induced eye inflammation.

However, the prolonged use of DEX may cause some systemic adverse effects such as muscle weakness, osteoporosis, cataracts, glaucoma, ecchymosis, insomnia, and skin changes (bruising/fragility/hirsutism) [11,12,13,14]. Some general side effects such as hypertension, hyperglycemia, and cognitive alterations have also been reported [15]. Although topical application can avoid such effects, the self-protective barriers of the eye and the tight junctions of the corneal and conjunctival epithelia allow only a small percentage of topically administered drugs to penetrate through ocular tissues [16], rendering limited drug availability to the anterior/posterior segments as well as corneal stroma [17]. Developing novel formulations that efficiently transport conventional ocular preparations across the cornea represents a major challenge. New ocular drug delivery carriers, such as mucoadhesive polymer-based nanoparticles, are needed to achieve effective ophthalmic drug levels. Chitosan (CS) polymer-based nanoparticles are believed to adhere to the surface of the eye for prolonged periods without causing significant irritation [18]. Furthermore, they have been shown to reversibly loosen corneal epithelial tight junctions and thereby improve the transcorneal flux of the applied drug [19].

Our previous studies exhibited less ocular bioavailability in terms of transcorneal permeation and aqueous humor drug concentration as compared to the HA-coated DEX-CSNPs [18]. Remarkably, prior evidence has demonstrated that DEX-loaded nanocarriers improved the efficiency of drug delivery applied to the eyes [20], with a high concentration of DEX accumulating on the ocular surface [18,21]. This in turn triggers transcorneal flux and delivery of DEX to the anterior and posterior segments of the eyes [22]. In that manner, DEX-loaded nanocarriers achieve controlled and constant delivery of DEX to the target site, ultimately reducing ocular inflammation [23].

The inherent properties of chitosan (CS) and hyaluronic acid (HA), such as biodegradability, biocompatibility, and susceptibility to enzyme-based hydrolysis and ocular safety make this a promising drug delivery platform [18,24,25]. Furthermore, HA has been shown to improve the proliferation (and hence regeneration) of corneal and conjunctival epithelial cells through direct interaction with CD44 receptors, which are increasingly expressed during ocular inflammation [17,26,27]. Additionally, the surfaces of HA and chitosan nanoparticles (CSNPs) bind to form an interfacial HA-CS complex, which has been shown to improve cellular targeting [28] and uptake via receptor-facilitated endocytosis [18].

The purpose of this study was to examine the therapeutic effects of DEX-loaded CSNPs in rabbits with LPS-induced uveitis. We evaluated the utility of uncoated and HA-coated DEX-CSNPs as a sustained ocular delivery vehicle to deliver DEX. The HA-coated DEX-CSNPs have been reported by many researchers, but in the present study, we focused on their pharmacodynamic application in EIU in rabbits. Furthermore, the transcorneal penetration of DEX on the excised rabbit cornea as well as the eye-irritation potential of the CSNPs, including ocular pharmacokinetics, were also assessed. 

## 2. Materials and Methods

### 2.1. Chemicals

Dexamethasone sodium phosphate (C_22_H_28_FNa_2_O_8_P; MW: 516.4 g/mol), hydrocortisone (C_21_H_30_O_5_; MW: 362.5 g/mol), low-molecular-weight chitosan (75–85% deacetylated) with viscosity average molecular weight of 50–190 k, sodium tripolyphosphate (sodium-TPP), and sodium dihydrogen phosphate were purchased from Sigma Aldrich (St. Louis, MO, USA). Glacial acetic acid was purchased from BDH Ltd. (Poole, UK). Hyaluronic acid (200 kDa) was obtained from Medipol SA (Lausanne, Switzerland). A Spectra/Por regenerated cellulose (RC) dialysis membrane with 12–14 kDa molecular weight cut-off was procured from Spectrum Laboratories, Inc. (Rancho Dominguez, CA, USA). Mannitol was purchased from Qualikems Fine Chem. Pvt. Ltd. (Vadodara, India). Methanol and acetonitrile (HiPerSolv CHROMANORM^®^ for HPLC) were purchased from BDH Prolabo^®^ (Leuven, Belgium). Purified water was obtained using a Milli-Q^®^ water purifier (Millipore, Molsheim, France). All other solvents were of HPLC grade, and the remaining chemicals were of analytical grade. LPS from *Escherichia coli* was purchased from ChemCruz (Santa Cruz Biotechnology, Inc. Dallas, TX, USA), and the ELISA kits were purchased from MyBiosource, Inc. (San Diego, CA, USA).

### 2.2. Preparation of CSNPs, Surface Coating, and Lyophilization of DEX-CSNPs

The chitosan nanoparticles (CSNPs) were prepared by the ionic-gelation method at physiologic pH range [29]. Self-aggregation of CS and Tripolyphosphate-Sodium (TPP-Na) resulted in ionic crosslinking, where TPP-Na acts as a cross-linker. The magnetic stirring (for 2–3 h at 700 rpm) at low *w*/*w* ratio of TPP and CS ratio produced stable NPs. The detailed method for the preparation of DEX-loaded CSNPs and their surface coating with HA was reported previously [24]. Briefly, CS was solubilized in 1% (*v*/*v*) acetic acid to obtain a concentration range of CS (0.2, 0.4, 0.6, and 1.0 mgmL^−1^). The DEX (10 mg) was dissolved in CS solution. The TPP was solubilized in Milli-Q^®^ water to get different concentrations (0.2, 0.4, 0.6, 0.8, and 1.0 mgmL^−1^) and the pH was maintained to 7.2 with 0.1 M sodium dihydrogen phosphate (NaH_2_PO_4_) buffer. Subsequently, TPP solution (6 mL) was added in CS solution (12 mL) at 1.5 mLmin^−1^ of rate of addition. For HA coating, 20 mg of CSNPs was suspended in 0.1 M acetic acid (2 mL) at pH 5. The suspension was added drop wise to 2 mL of HA containing (0.5, 1, 2, 5, 10, and 20 mgmL^−1^) 0.1 M acetic acid solution. The process was performed at magnetic stirring (1000 rpm for 30 min). Thereafter, the nanosuspension was ultra-filtered against purified water through the dialysis membrane [30,31]. The suspensions of DEX-CSNPs and HA-coated DEX-CSNPs were lyophilized with and without mannitol (2.5%, 5%, and 7.5% *w*/*v*) [32,33] and then stored at 25 °C for further characterization. The nanosuspensions of CSNPs were filtered through the Millipore^®^ syringe filters (450 µ), frozen at −80 °C, and lyophilized by FreeZone-4.5 Freeze Dry System (Labconco Corporation, Kansas City, MO, USA). The lyophilization was performed with and without mannitol at varying concentrations (2.5%, 5%, and 7.5%, *w*/*v*) as lyoprotectant. The lyophilized products were stored as mentioned above.

### 2.3. Physical and Physicochemical Characterizations

The physical characterizations including the size, distribution, polydispersity-index (PDI), and zeta potentials (ZP) of the DEX-CSNPs (HA-coated and uncoated, with and without mannitol) were determined by dynamic light scattering (DLS) using Zetasizer Nano-ZS (Malvern Instruments Ltd., Worcestershire, UK).

The morphologies of HA-coated and uncoated DEX-CSNPs were characterized by transmission electron microscopy (TEM). Concisely, the nanosuspensions were sonicated for 5 min prior to grid preparation. A copper grid (300 meshes) with carbon type-B support film (manufactured by Ted-Pella Inc. Redding, CA, USA) was kept on butter paper. One drop of the CSNPs suspensions (previously sonicated for 5 min) was put separately on the grid and left for 15 min to settle down the NPs. The grid was left overnight to dry. The dried grid was then mounted in the sample holder of the machine, and the shape of the NPs was investigated under JEM-1010, TEM (JEOL, Tokyo, Japan). The machine operated at 80 kV (accelerating voltage) and 60,000- to 150,000-times magnification power at room temperature [34].

Drug encapsulation efficiency (EE) and loading capacity (DL) were estimated indirectly by measuring the free drug concentration of DEX via an ultra-performance liquid chromatography coupled with the ultraviolet detection (UPLC-UV) method [24,35]. Briefly, the Waters Acquity H-Class UPLC system coupled with a Waters TUV Detector by Acquity (Waters, Milford, MA, USA) for the analysis of DEX was used. Elution of DEX was completed on a Acquity UPLC BEH^TM^ C_18_ Column (1.7 µm, 2.1 × 50 mm) that was maintained at ambient temperature. The mobile phase (60/40 acetonitrile and water, where the pH of water was adjusted to 3.2 with O-phosphoric acid) was isocratically pumped at 0.14 mL.min^−1^ flow rate, and the volume of injection was 10 µL. The EMPOWER software was used for data acquisition, processing, as well as to control the UPLC system.

The transparency of the prepared nanosuspensions (coated and uncoated) was determined by visual observation under light against a black and white background. The pH was checked using a pH-meter (MP-220; Mettler Toledo, Switzerland), and the refractive index was estimated by an Abbe Refractometer (model DR-A1, ATAGO, Inc., Bellevue, Washington, USA). The viscosity of nanosuspensions was measured using a cone and plate viscometer (Physica Rheolab, Austria) with an MK-22 spindle. The above parameters were evaluated initially and after three months storage at 25 ± 2 °C, as reported previously [36,37,38].

### 2.4. In Vitro Drug Release

The dialysis membrane method was employed for the in vitro drug release experiment [39]. After maintaining the isotonicity by mannitol, an equivalent amount of NPs and DEX-solution in triplicate, containing 0.1% (*w*/*v*) of drug (i.e., 1.0 mg/mL), were placed in dialysis bags (MWCO 10–12 kDa) and both ends were tightly closed. The filled bags were put in beakers containing 50 mL of simulated tear fluids (STF). The whole assembly was placed in a water bath (shaken at 50 rpm and maintained at 35 ± 0.5 °C just to mimic the ocular surface temperature). Samples were withdrawn at predetermined time points and the same amount of fresh STF (maintained at 35 ± 0.5 °C) was replaced after each sampling to maintain the sink conditions. The withdrawn samples were centrifuged for 10 min at 13,000 rpm and 4 °C, supernatants were collected (diluted with STF, whenever needed), and the concentration of released DEX was analyzed using UPLC-UV as described previously [24,35,40]. A calibration curve (y = 10984*x* – 639.32), *R*^2^ = 0.999, was used to calculate the DEX concentration. The cumulative amounts of drug released (%DR) was calculated using Equation (1) and plotted against time (h).

(1) $$\%\;DR=\frac{Conc.\;\left(µgmL^{-1}\right)\times Dilution\;factor\times Volume\;of\;STF\;\left(mL\right)}{Initial\;dose\;\left(\mu g\right)}\times 100$$

### 2.5. In Vivo Animal Study

Thirty male New Zealand Albino rabbits (2.0–3.0 kg) were acquired from the College of Pharmacy (Animal Care and Use Center, King Saud University, Riyadh, Saudi Arabia). The animal experiments were performed as per the Association for Research in Vision and Ophthalmology (ARVO) statement regarding the use of animals in ophthalmic and vision research, and they were approved by the Institutional Animal Care and Use Committee of King Saud University (SE-19-90). The drug-loaded CSNPs were subjected to in vivo ocular experiments based on the results of the physicochemical characteristics, in vitro drug release study, and permeation parameters.

#### 2.5.1. Ocular Irritation Study

The drug-loaded CSNPs intended for topical ocular application were cryoprotected by mannitol (2.5%, *w*/*v*), freeze-dried, and sterilized by UV-radiation. The formulations were exposed to UV-light at 254 nm wavelength for 2 h [41,42]. The formulations were reconstituted in sterile water for injection before ocular administration. Six rabbits were divided in two groups (*n* = 3). The eye irritation test was performed as per Draize’s protocol for rabbits [43]. We instilled the sterilized formulations into rabbit eyes three times/day for 10 days and visually observed them throughout the experiment. The level of eye irritation was judged by observing the animals’ uneasiness and assessing signs/symptoms in the cornea, conjunctiva, and eyelids according to previously reported scoring systems [44].

#### 2.5.2. Transcorneal Permeation

After a washout period of three weeks, five rabbits (previously utilized for ocular irritation experiments) were sacrificed to excise ten corneas. Among these, nine corneas were used for transcorneal permeation study by the double-jacketed automated transdermal diffusion cell-equipped sampling system (SFDC-6; Logan, NJ, USA). The detailed methodology was as previously reported [18]. The cross-section of the cornea measured 0.636 cm^2^. Phosphate buffered saline (PBS; 6.9 mL, pH = 7.4) was used as the release medium, and the initial drug concentration was 500 µg∙mL^−1^. The study was performed in triplicate for 6 h and the amount of permeated DEX was analyzed by UPLC-UV [35]. Permeation parameters such as steady-state flux (*J*, µgcm^−2^s^−1^) and apparent permeability (*P*app, cms^−1^) were determined by Equations (2) and (3).

(2) $${J\;(µ\mathrm{gcm}}^{-2}s^{-1})=\;dQ/dt$$

(3) $${P\mathrm{app}\;(\mathrm{cms}}^{-1})=\;J/C_{0}$$

where *Q* is the quantity of DEX that passes through the cornea, *t* is the exposure time, and *C*_0_ is the initial DEX concentration (µg∙mL^−1^) in the donor compartment of the Franz diffusion cell.

#### 2.5.3. Ocular Pharmacokinetics

Nine animals were divided into 3 groups (*n* = 3). As per the directive of the Association for Research in Vision and Ophthalmology (ARVO) for animal use in ophthalmic and vision research only one eye (either right or left) should be used for the experimental purpose. Therefore, only the left eye of all rabbits was treated. The concentration of DEX in AH was determined after the instillation of sterilized formulations such as DEX-CSNPs (group-A), HA-coated DEX-CSNPs (Group-B), and DEX-aqueous solution (DEX-AqS; Group-C). In addition, AH was collected at different time intervals, and its DEX concentration was analyzed by UPLC-UV as previously reported [18]. A non-compartmental approach was used for determining pharmacokinetic parameters. The area under the curve to the last measurable concentration (AUC_0–t_), area under the curve to infinity (AUC_0–inf_), maximum concentration (C_max_), time to C_max_ (t_max_), and half-life (t_1/2_) were computed using *PK*-Solver software (Nanjing, China) in Microsoft Excel 2013 [18,45]. The paired *t*-test (GraphPad Software, Inc., San Diego, CA, USA) was utilized to compare the obtained pharmacokinetic parameters; *p*-value < 0.05 was considered statistically significant.

#### 2.5.4. Effect of DEX-CSNPs on LPS-Induced Ocular Inflammation

##### Study Design and Animal Model of Experimental Uveitis

Fifteen rabbits were divided in 5 groups (*n* = 3). Rabbits in Group-1 (normal control group) received 5% mannitol (vehicle). Group-2 was injected with LPS. Group-3 received DEX-AqS. Group-4 was treated with DEX-CSNPs and Group-5 was treated with HA-coated DEX-CSNPs. All groups except Group-1 received intravitreal injections of LPS (20 µL; 100 ng) in both eyes to induce uveitis [46,47]. Topical anesthesia was applied by administering one drop of 0.5% Proparacaine Hydrochloride. After retracting the upper lid, 100 ng (20 μL, dissolved in water for injection) of the endotoxin was injected intravitreally with a 29-gauge needle. After the induction of uveitis, the sterilized formulations were instilled topically into the animals’ left eye 3 times a day for 3 days. Seventy-two hours after the induction of uveitis, AH was sampled for cell count, protein, interleukin-6 (IL6), myeloperoxidase (MPO), nuclear factor kappa B (NF-κB)-DNA binding activity, and estimated TNF-α [20]. Rabbits were re-examined for clinical signs of uveitis and then sacrificed. Animals’ eyes were enucleated for histopathologic examination [48].

##### Grading System for Ocular Inflammation

The clinical signs of ocular inflammation were graded on a scale of 0 to 4 according to a previously reported scoring system [49] as follows: no inflammatory reaction (= 0), discrete inflammatory reaction (= 1), moderate dilation of the iris and conjunctival vessels (= 2), intense iridial hyperemia with flaring in the anterior chamber (= 3), intense iridial hyperemia with intense flaring in the anterior chamber and the presence of fibrinoid exudates in the papillary area (= 4). Grading was performed 24 and 72 h post intravitreal injection of LPS [47,48].

##### Aqueous Humor (AH) Sampling

A combination of Ketamine Hydrochloride (20–40 mg∙kg^−1^ b. wt.) and Xylazine (1–2 mg∙kg^−1^ of body weight) was intravenously injected into the rabbits’ marginal ear veins to induce anesthesia. The Proparacaine Hydrochloride (0.5%, *w*/*v*) was instilled in the eyes to facilitate general anesthesia. A 29-gauge needle was used to remove approximately 50–100 μL of AH from the anterior chamber of the eyes while taking care not to injure the iris, lens, and other areas of the eyes. 

##### Total Cell Count

Approximately 50 µL of AH was suspended in 50 µL of Turk’s stain solution. Cells were counted using a hemocytometer with the aid of a light microscope. Afterward, the number of cells per milliliter of AH was calculated [50,51]. Cell counting was performed on the day of AH sampling.

##### Estimation of Total Protein and Inflammatory Cytokines (TNF-α and IL6)

The Estimation of total protein in AH samples was performed according to Lowry’s method [52]. Briefly, AH (10 μL) samples were diluted with 990 μL of 1 N NaOH and reacted with 4 mL of copper reagent. After 10 min, 500 μL of Folin’s reagent was added to each sample and the solutions were vortexed. Then, the samples were stored for 30 min in the dark. Absorbance was recorded with a spectrophotometer at 620 nm. Bovine serum albumin (BSA) was used as the standard for calculating the protein content of the samples. All estimations were performed in triplicate. Protein estimation was performed on the day of AH sampling. The levels of TNF-α and IL6 in AH were determined by using a commercially available ELISA kit per the manufacturer’s instructions [53].

##### Western Blot Assay

Frozen eye tissues were homogenized in a 0.5% (*w*/*v*) hexadecyltrimethylammonium bromide solution, solubilized in 0.01 M potassium phosphate buffer (pH = 7), and centrifuged at 6000 rpm for 30 min at 4 °C. The concentration of protein in tissues was measured by Lowry’s method [52]. Tissue lysates (25 µg/well) were loaded in 10% Mini-Protean TGX Gels (Bio-rad, Hercules, CA, USA), underwent electrophoresis, and were transferred to a PVDF membrane (Bio-rad, Hercules, CA, USA). The membrane was then blocked, and, with 5% (*w*/*v*) skimmed milk, prepared in Tris-buffered saline and Tween 20 (TBS-T). The membrane was then incubated with primary antibodies prepared based on the manufacturer’s recommendation overnight at 4 °C. Membranes were then washed and incubated with the suitable secondary antibody, horseradish peroxidase-coupled anti-rabbit or anti-mouse antibody. Bands were visualized using the Western Bright ECL Kit for 5000 cm^2^ Membrane and Blue Basic Autoradiography Film (Bioexpress, Kaysville, CA, USA) [54].

##### Histopathological Evaluation

The eyes of sacrificed rabbits were enucleated 72 h post intravitreal injection of LPS and stored in 10% (*v*/*v*) formaldehyde solution. Sections (approximately 4 to 5 µm thick) were cut and stained with Hematoxylin and Eosin stains (H&E) and evaluated using light microscopy.

### 2.6. Statistical Analysis

All results were expressed as mean of three measurements with standard deviation (Mean ± SD). The statistical analysis was performed using GraphPad Prism V 5.0 (GraphPad Software Inc., San Diego, CA, USA). All parameters were compared using one-way ANOVA. The paired *t*-test (GraphPad Software Inc., San Diego, CA, USA) was employed to compare the obtained parameters, considering the *p*-value *(p* < 0.05) as statistically significant.

## 3. Results and Discussion

### 3.1. Development of DEX-CSNPs and Their Coating

CSNPs were prepared by ionic gelation, wherein self-aggregation of CS and sodium-TPP resulted in ionic crosslinking. Continuous magnetic stirring (at 500 rpm for 2 h) at low TPP: CS weight ratios produced stable NPs. The TPP: CS ratio of 0.4:0.6 (mg/mL) produced NPs of an optimal size suitable for ocular application. They had high EE, DL, and appropriate drug release properties that made them ideal for HA-coating and further characterization [24,40].

The optimum CSNPs were coated with a 2% (*w*/*v*) solution of HA in diluted acetic acid according to the previous reports [24,31]. We coated CSNPs with HA because it enhances cellular targeting and interacts with CD-44 receptors to regenerate corneal and conjunctival epithelia [17], which in turn supports receptor-mediated transportation and hyaluronan biodegradation [55]. Hyaluronic acid-coating reversed the surface charge of NPs from highly positive to negative. This could be attributed to the effective adsorption of negatively charged HA molecules to positively charged CSNPs. A reversal in surface charge can result in a high negative charge density around HA-coated CSNPs, which in turn increases their hydrodynamic diameters [56]. Furthermore, HA-coating also was able to protect against the pH-dependent endosomal-mediated disruption of CS [30]. Thus, HA-coating of CSNPs is key for CSNP stability over time, particularly, under low pH conditions within the lysosomes.

### 3.2. Characterization of HA-Coated DEX-CSNPs

**Figure 1:**
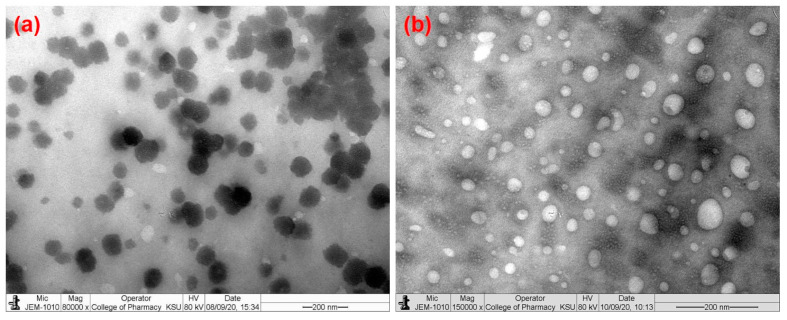
Transmission electron micrographs of DEX-loaded CSNPs: (**a**) uncoated; (**b**) HA-coated.

**Figure 2:**
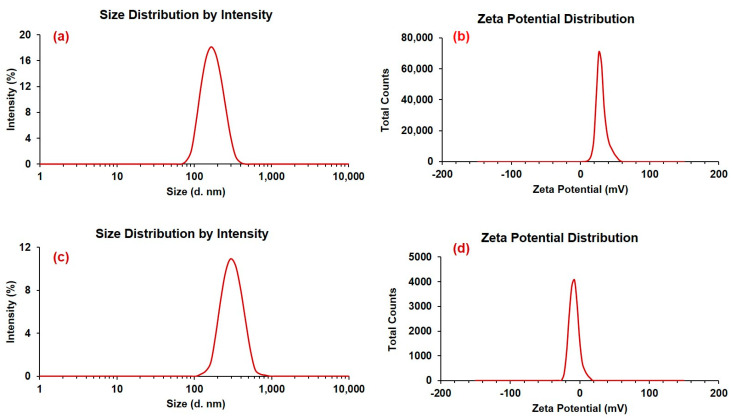
Particle size and zeta potential distributions of DEX-loaded CSNPs; (**a**,**b**) for uncoated; (**c**,**d**) for DEX-loaded CSNPs coated with HA.

The representative TEM images (Figure 1a,b) indicated that DEX-loaded CSNPs were evenly distributed and did not aggregate. They were found to have solid, dense, spherical morphology with a smooth surface (in the case of HA-coated ones), while the surfaces of uncoated CSNPs were slightly rough, which was also reported previously [57]. Apart from the surface modification, HA-coating increased the particle size, which was further confirmed by DLS measurement as represented in Figure 2. Here, Figure 2a,b represents the particle size and zeta potential distributions of the DEX-loaded CSNPs (uncoated), whereas Figure 2c,d represents the particle size and zeta potential distributions of the DEX-loaded CSNPs coated with HA.

Particle size, polydispersity index (PDI), and zeta potentials are important parameters for preventing eye irritation as well as for prolonging drug retention in the conjunctiva and cornea. Particles must be small (ranging from a few nm to 900 nm), since larger particulates may cause ocular irritation and discomfort and possibly negatively affect patient compliance [58]. In our study, the size of CSNPs was 310.4 ± 12.4 nm before lyophilization and without HA coating, and 368.5 ± 14.4 nm after lyophilization and HA coating. Because human eyes can tolerate particulates up to 10 µm in diameter [58,59], our system of HA-coated CSNPs would be a good candidate for the ocular delivery of DEX.

Zeta potential and PDI values are summarized in Table 1. High negative or positive zeta potentials would probably lead to a stable colloidal solution, where electrostatic repulsion prevents NP aggregation. Small PDI values also are indicative of stable dispersion and unimodal distribution of CSNPs in the dispersion medium (Table 2). The CSNPs produced by magnetically stirring TPP and CS at a ratio of 0.4:0.6 (mg/mL) for 2 h at 500 rpm had good EE and DL. In order to evaluate the effect of drug concentration on EE and DL, varying amounts of DEX (5, 10, and 15 mg) were added to the DEX-CSNPs formula. We found that 10 mg DEX was optimal for producing DEX-CSNPs with high EE and DL and for which all the physical characteristics were appropriate for ocular application. The values of the obtained parameters are summarized in Table 2.

The physicochemical characteristics, including clarity, refractive index, pH, and viscosity of the CSNP suspensions were deemed appropriate for ocular application (Table 3). That is, the pH values of CSNP suspensions, which remained virtually unchanged throughout the storage period, were suitable for ocular use (approaching to the normal physiological pH of ocular surfaces in humans, i.e., 7.1 ± 1.5) [60]; and the observed refractive indexes were similar to that of tear fluid. Thus, we anticipate that the formulations would not impair vision and would be comfortable. The viscosity of the nanosuspension affects the proper instillation of ophthalmic medications as well as the ease of sterilization (if by filtration). The observed viscosity of the two nano-formulations was within the range of optimal viscosity (20–30 mPa.s) for ocular preparations [61,62]. Hence, there was no chance of eye discomfort because of blurred vision and foreign particles, and in turn, there was no risk of the elimination of preparations due to reflex tears and frequent eyelid blinking [62,63,64]. Therefore, the formulations could be retained for prolonged periods without impairing the vision.

### 3.3. In Vitro Drug Release

**Figure 3:**
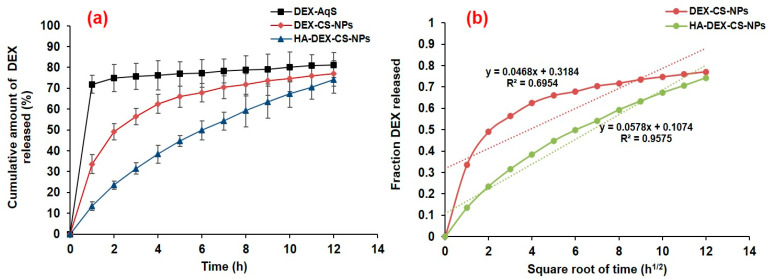
Cumulative amount of drug released versus time profile (**a**) and Higuchi’s square root plot (**b**). The data were expressed as the mean of three measurements with standard deviations (Mean ± SD, *n* = 3).

Weighed CSNP samples with equivalent amounts of DEX (0.1%, *w*/*v*) were used for in vitro release experiments based on encapsulation and initially prepared concentrations of drug and excipients. The in vitro release experiment demonstrated an initial burst release of DEX from the uncoated CSNPs lasting ~3 h, after which there was a sustained release for up to 12 h. On the other hand, there was a sustained release of DEX from the HA-coated CSNPs. We noted that 33.62% of the drug was released at 1 h and around 56.32% at 3 h from the uncoated CSNPs in the rapid release phase, while it was only 13.49% at 1 h and went to 31.54% at 3 h from the HA-coated NPs. The release profile (Figure 3a) indicated slow drug release from the HA-coated NPs, which must be due to the HA-coating of the DEX-loaded CSNPs [24,40]. Although the total amount of released drug from both the NPs was almost comparable, the pattern of drug release from the HA-coated NPs was sustained and consistent over 12 h. In addition, the sustained release of DEX from HA-coated NPs can be explained by Higuchi’s square root plot (Figure 3b). It represents the release rate plots for the diffusion of DEX from the CSNPs, where the fraction of the released drug was plotted against the square root of time. The increase in the fraction of DEX released from the HA-coated CSNPs was practically linear (with *R*^2^ = 0.957) against the square root of time (h^1/2^) as compared to uncoated CSNPs (*R*^2^ = 0.695), which justifies the sustained-release property of HA-coated CSNPs rather than that of the uncoated one [39].

The initial rapid release of DEX from the uncoated CSNPs could be attributable to a desorption phenomenon, i.e., the rapid dissolution and diffusion of the surface-adsorbed loosely bound drug from the surface of NPs [65], while the release rate of DEX from HA-coated NPs was slower during the initial hours due to the HA-coating. One explanation is that the HA-coating hindered the rapid dissolution and diffusion of the drug from the CSNP core in the release medium (STF). The slow and sustained release of DEX from the HA-coated NPs is owing to the changes in the release mechanism (including liberation and diffusion of drug from the polymer matrix). Another possibility for such an outcome may be due to the polymer degradation in STF or even the combined effects of both drug diffusions from the polymer matrix and polymer degradation in STF.

### 3.4. Transcorneal Permeation

**Figure 4:**
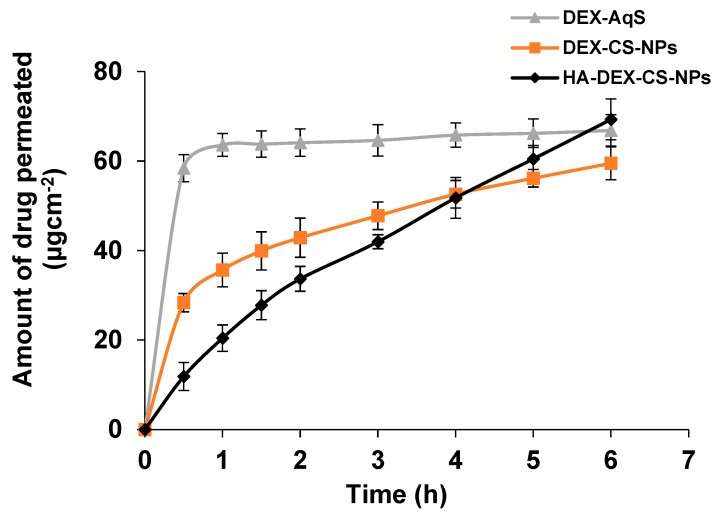
Transcorneal permeation of DEX from CSNPs and DEX−aqueous solution (0.1%, *w*/*v*). The data were presented as the mean of three readings with standard deviations (Mean ± SD, *n* = 3).

The permeation flux and *P*_app_ values of different formulations were calculated (Table 4). The uncoated CSNPs were able to permeate around 28.4 µgcm^−2^ of drug, while it was only 11.8 µgcm^−2^ for the HA-coated CSNPs at 30 min. The HA-coated CSNPs achieved a sustained transcorneal permeation of DEX starting from 30 min until 6 h (Figure 4), which reached a maximum of 69.32 µgcm^−2^ at 6 h. There was around a 4.7-fold and 10.1-fold enhanced flux for uncoated and HA-coated CSNPs, respectively, as compared to DEX-AqS. Due to high mucoadhesiveness and HA interaction with hyaluronan receptors on corneal surfaces, our nano-formulation exploits this property of surface targeting [28] that could potentially enhance cellular uptake through receptor-mediated endocytosis [18]. This could explain the improved permeation of the HA-coated CSNPs compared to the uncoated CSNPs.

A neutral pH plays an important role in DEX permeation through the cornea. The first pK_a_ of DEX is 1.89, at which point the ratio of neutral: monoanion is 50:50. At the second pK_a_ (6.4), the monoanion: dianion ratio is 50:50. Because DEX has maximum mobility at pH 7, the second pK_a_ provides high water solubility with sufficient buffering capacity to the formulations for ocular use [66]. In our study, the pH of CSNP suspensions was almost neutral (equivalent to that of tears), whereby a large amount of DEX remained in the unionized state to promote high corneal permeation. The observed additional DEX permeation in the initial hours of the experiment (Figure 4) could be potentially due to the large fraction of unionized DEX at the neutral pH (7.11 ± 0.12) of DEX-AqS [18,67].

### 3.5. Ocular Irritation

The ocular irritation of CSNPs suspensions in rabbit eyes was assessed against NaCl solution (0.9%, *w*/*v*). We have shown in a previous study that recurrent instillation of uncoated and HA-coated CSNPs resulted in slight eye irritation in some rabbits [18]. In contrast, none of the animals in our current study displayed acute or long-term signs of discomfort (Grade 0), which might be due to the immune variability of the animals. Moreover, irritation levels for the conjunctiva, cornea, and eyelids were Grade 0 among rabbits receiving coated and uncoated DEX-CSNPs. The results of this experiment support that DEX-CSNPs are safe and nonirritating for ocular use.

### 3.6. Ocular Pharmacokinetics

**Figure 5:**
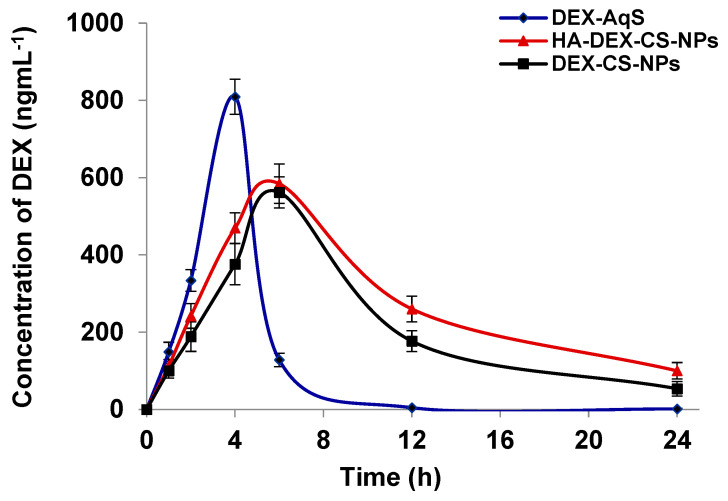
Aqueous humor (AH) drug concentration-time profile of DEX following topical ocular instillation of different formulations containing DEX in rabbits. The data were presented as the mean of three readings with standard deviations (Mean ± SD, *n* = 3).

The previously validated UPLC-UV method is effective for the analysis of DEX in aspirated AH samples after the topical application of DEX-containing formulations [18]. The measured concentrations of drug in AH samples at different time points are represented in Figure 5, and the pharmacokinetic parameters calculated using PK-Solver are summarized in Table 5.

The concentrations of DEX in the AH samples were easily quantified for up to 6 h in Group-C (DEX-AqS group); but afterward, the drug was not detectable, demonstrating the relatively faster precorneal loss of DEX from aqueous solution. In contrast, DEX was easily quantified for up to 24 h in animals treated with uncoated (Group-A) and HA-coated (Group-B) drug formulations. The ocular bioavailability of DEX was significantly (*p* < 0.005) higher in the DEX-CSNPs formulations compared to DEX-AqS. The difference in AUC_0–24h_ was approximately 1.87- and 2.36-fold greater in uncoated and HA-coated DEX-CSNPs compared to that of DEX-AqS, respectively. The biological t_1/2_ of DEX from uncoated and HA-coated DEX-CSNPs was 2.49- and 3.36-fold higher, while the C_max_ of the drug from uncoated and HA-coated DEX-CSNPs was 1.44- and 1.38-fold lower than that of DEX-AqS, respectively. Mean residence time to infinity (MRT_0–inf_) of the drug in the ocular area was 2.47- and 3.15-fold greater for uncoated and HA-coated DEX-CSNPs as compared to DEX-AqS. The strong mucoadhesive nature of CS and HA is known to support ocular bioadhesion of the DEX-loaded NPs [17]; hence, extending drug retention in the eyes thereby enhances drug availability to ocular tissues [68]. Similarly, we believe that the strong interaction between HA and CD44/hyaluronan receptors on the epithelial surface of ocular tissues is responsible for the improved pharmacokinetic parameters of the HA-coated CSNPs.

Indeed, our computed pharmacokinetic parameters suggest the bioadhesion of DEX-CSNPs to the corneal and conjunctival epithelium, which enhanced ocular retention and maintained a high transcorneal DEX flux exceeding that of DEX-AqS. In the case of HA-coated DEX-CSNPs, and consistent with previous reports, we believe that such a response is due to the direct interaction with hyaluronan receptors on corneal and conjunctival epithelia [18,27], leading to improved drug retention of the HA-coated DEX-CSNPs on ocular surfaces and an associated transcorneal flux and permeability of the drug.

The HA is known to improve cellular targeting and accelerate cellular uptake of the HA-coated NPs through receptor-mediated endocytosis [18,69]. The high ocular bioavailability of DEX in the HA-coated DEX-CSNPs might have been reinforced by the phagocytic propensity of conjunctival and corneal epithelial cells for HA-coated CSNPs [18,68]. Since the pharmacokinetic parameters of DEX preparations had low variability and were consistent throughout the in vivo, this suggests promising potentials of CSNPs in the topical or intravitreal delivery of DEX to the eyes.

### 3.7. Ocular Pharmacodynamics

**Figure 6:**
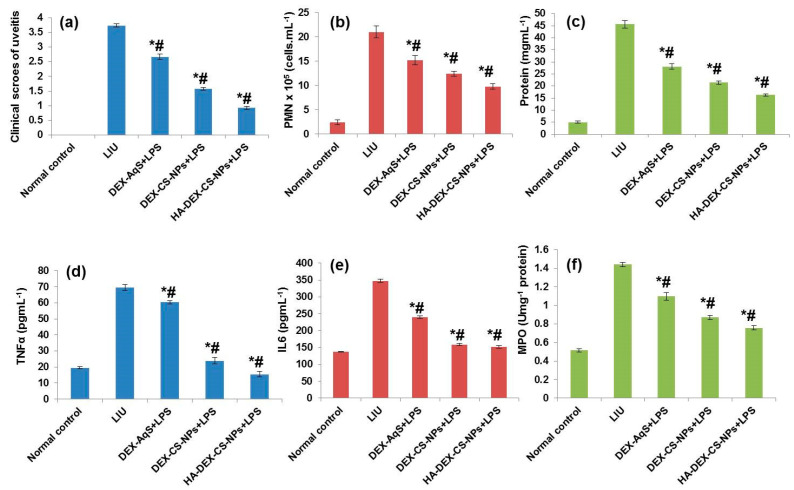
Effects of DEX treatment on LPS-induced uveitis in rabbits: (**a**) clinical scores of uveitis; (**b**) infiltrations of polymorphonuclear (PMN) cells; (**c**) protein concentrations; (**d**) tumor necrosis factor-α; (**e**) interleukin-6; (**f**) and myeloperoxidase (MPO) in the AH of rabbits after intravitreal injection of LPS. Compared to normal control (*)/to LPS control (#), but the effect did not reach significance (*p* > 0.05). All the data were expressed as the mean of three measurements with standard deviations (Mean ± SD, *n* = 3).

Intravitreal LPS injections induced inflammatory reactions with a marked cellular flare in all LPS-treated rabbit groups. Treatment of LPS-induced uveitis (LIU) with DEX-AqS, DEX-CSNPs, and HA-coated DEX-CSNPs significantly suppressed ocular inflammation in rabbits, as evidenced by visual inspection (i.e., grading in a blinded fashion 24 h after LPS injection). The clinical signs of ocular inflammation, on a scale of 0 to 4 according to a previously published scoring system [49] are presented in Figure 6a. Lipopolysaccharide treatment induced severe inflammation, such that the clinical score for ocular inflammation (3.732 ± 0.053; 100%) was several folds higher in LIU rabbits than in the normal control group. Ocular treatment with DEX-AqS, DEX-CSNPs, or HA-coated DEX-CSNPs significantly reduced those clinical scores to 2.67 ± 0.085 (28.45%), 1.578 ± 0.048 (57.71%), and 0.93 ± 0.053 (75.08%), respectively. Importantly, the clinical scores clearly demonstrated that improving the bioavailability and sustained-release characteristics of DEX help in suppressing ocular inflammation. Topical steroids have been highly effective at mitigating ocular inflammation in several uveitis models [70,71]. In our study, intravitreal injections of LPSs increased cellular infiltration of polymorphonuclear (PMN) cells and monocytes into the AH of rabbits by 775% (LIU). However, this was significantly suppressed through DEX treatment to 533.33% with DEX-AqS, 416.67% with DEX-CSNPs, and 308.33% with HA-coated DEX-CSNPs. Accordingly, our findings demonstrate that HA-coated DEX-CSNPs might exhibit improved clinical outcomes as manifested by suppression of PMN infiltration to the aqueous humor (AH). Turk’s staining further illustrated the level of cellular infiltration in LIU rabbits, demonstrating that DEX formulations inhibit the augmentation of cellular infiltrations (Figure 6b). To confirm such an improved protective effect of our HA-coated DEX-CSNP platform compared to other formulations and DEX-AqS, we quantified the protein levels in AH of the formulations studied; HA-coated DEX-CSNPs were the strongest inhibitors of ocular inflammation, as evidenced by reductions in cellular infiltration and clinical scores. To investigate the mechanism by which the formulations inhibited inflammation, we evaluated the effect of various DEX formulations on AH protein concentrations, which we estimated using Lowry’s method [52]. As shown in Figure 6c, the protein concentration in the AH of LIU rabbits was 801.47% (5.05 ± 0.40 to 45.61 ± 1.63 mg∙mL^−1^) higher than that of normal rabbits, owing to the cellular infiltration, cytokines, and chemokines at the site of ocular inflammation. The effectiveness of treatment in order of the percentage reduction in the protein concentration with respect to that of untreated LIU rabbits was as follows: HA-coated DEX-CSNPs (64.3%; 45.61 ± 1.63 to 16.27 ± 0.46) > DEX-CSNPs (52.9%; 45.61 ± 1.63 to 21.48 ± 0.66) > DEX-AqS (38.4%; 45.61 ± 1.63 to 28.08 ± 1.09) > LIU (0%; 45.61 ± 1.63 to 45.61 ± 1.63) (Figure 6c). These results suggest that HA-coated DEX-CSNPs and uncoated DEX-CSNPs significantly attenuated ocular inflammation as is evident from the reduction in protein concentrations in AH.

In the LIU model for acute inflammation, researchers have shown that PMN cells, neutrophils, and monocytes migrate from the iris venules and infiltrate the surrounding ocular tissues [72,73]. Our study also evaluated the levels of TNFα, IL-6, and MPO following intravitreal injection of LPS and in response to the different drug formulation. Our results demonstrated that LPS treatment induced the influx of all cytokines (i.e., TNFα, IL-6, and MPO). However, the drug formulations have suppressed these levels as Figure 6d–f show, which were all statistically significant. The effectiveness of treatment based in the percentage reduction in TNF-α levels with respect to that of untreated LIU rabbits was as follows: HA-coated DEX-CSNPs (77.7%) > DEX-CSNPs (65.6%) > DEX-AqS (13.2%). The effectiveness of treatment in order of the percentage reduction in IL6 levels with respect to that of the untreated LIU rabbits was as follows: HA-coated DEX-CSNPs (56.3%) > DEX-CSNPs (54.3%) > DEX-AqS (31.2%). Also, the effectiveness of treatment in order of the percentage reduction in MPO levels with respect to that of the untreated LIU rabbits was as follows: HA-coated DEX-CSNPs (47.4%) > DEX-CSNPs (39.4%) > DEX-AqS (23.7%). Thus, the results indicate that the DEX formulations have significantly ameliorated ocular inflammation by inhibiting the release of cytokines and reducing cellular infiltration. These results might be owed to the superior pharmacokinetic parameters exhibited by the DEX-CSNP, including the drug bioavailability and sustained-release efficacy.

**Figure 7:**
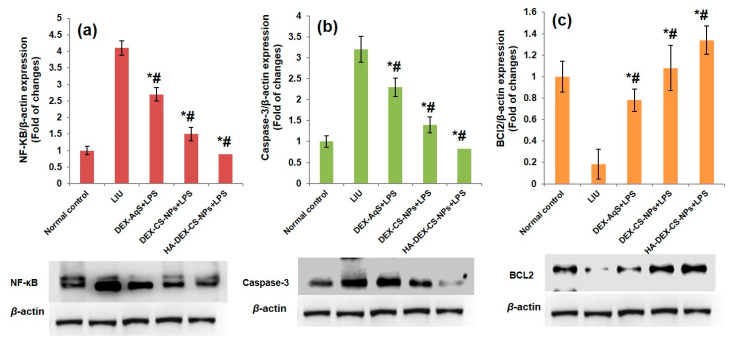
Effect of DEX treatment on LPS-induced uveitis alteration in inflammatory and apoptotic markers in uveal tissues of LIU rabbits. Western blot analysis of apoptotic markers: (**a**) nuclear NF-κB [p65], (**b**) Caspase-3, and (**c**) BCl2, compared to normal control (*)/to LPS control (#), but the effect did not reach significance (*p* > 0.05). All the data were expressed as the mean of three measurements with standard deviations (Mean ± SD, *n* = 3).

Lipopolysaccharides are known inducers of redox-sensitive transcription factor NF-κB, which plays a key role in eliciting a cascade of pro-inflammatory genes, such as TNF-α, IL-1β, IL-6, and COX-2, in different inflammatory conditions, including uveitis [74,75,76]. Under physiological conditions, the p65 subunit of NF-κB is bound with its inhibitor to form a trimetric complex (IκB-NF-κBp50/p65). Upon exposure to LPS, for instance, p65 is released to translocate to the nucleus to induce gene transcription. Exposure of THP-1 monocytes to LPS for 24 h enhances p65 protein levels in the cytosol and nucleus [77] and causes the number of NF-κB p65-positive cells in iris ciliary bodies to gradually increase over a period of 3–24 h [78,79]. In our study, NF-κB p65 proteins were overexpressed in the LIU group (Group-2) compared to the normal control group (Group-1) at 24 h after the LPS injection. Treatment with DEX-CSNPs and HA-coated DEX-CSNPs inhibited NF-κB p65 expression and alleviated ocular inflammation in the LIU rabbits as illustrated in Figure 7. Lipopolysaccharides induce TNF-α–dependent apoptosis in inflammatory tissues of the eye [80]. In turn, TNF-α induces host cell destruction by stimulating caspase-3, a downstream cysteine proteinase, through various apoptotic pathways [81,82]. Immunoblot analysis revealed that expression of the pro-apoptotic protein caspase-3 was enhanced and expression of the anti-apoptotic B-cell lymphoma 2 (BCL2) protein was reduced in LIU tissues compared to that of normal control tissues. Treatment with DEX formulations significantly mitigated the extent of apoptosis by down-regulation of caspase-3 and up-regulation of BCL2 proteins. The effectiveness of the treatment in order of the extent of reduction in apoptosis was as follows: HA-coated-DEX-CSNPs > DEX-CSNPs > DEX-AqS. These results further established that HA-coated DEX-CSNPs and DEX-CSNPs significantly attenuate LPS-induced apoptotic injuries in uveal tissues, as illustrated in Figure 7a–c.

**Figure 8:**
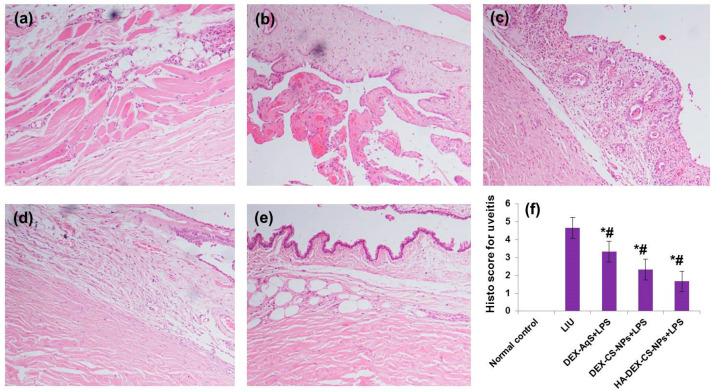
Effects of DEX treatment on the histopathological changes of intraocular inflammation of LIU rabbits. (**a**) Infiltrations of PMN cells were displayed in normal control rabbits. (**b**) Highest PMN cells infiltrated the extravascular uveal tissue in the vehicle + LIU rabbits. (**c**) Slight reduction in PMN cells infiltrated the extravascular uveal tissue in the DEX-loaded CS-NPs + LIU rabbits. (**d**) Moderate reduction in PMN cells infiltration in the extravascular uveal tissue in the DEX-loaded CS-NPs + LIU rabbits. (**e**) Maximum reduction in PMN cells infiltration in the extravascular uveal tissue in the DEX-loaded HA-coated CS-NPs + LIU rabbits. (**e**) Histopathological scores of LIU rabbits. Compared to normal control (*)/to LPS control (#), but the effect did not reach significance (*p* > 0.05). The data in (**f**) were expressed as mean with standard deviations three measurements. Tissues were stained with hematoxylin and eosin and viewed under 200× magnification.

Finally, histological examination of the LIU group (Group-2) showed substantial cell infiltration, primarily into the anterior chamber, compared to that of the normal control group. Histological scoring and pathology revealed that amelioration of LIU reduced the inflammatory cell infiltration into the anterior chamber of cells. The effectiveness of the treatment in order of the extent of reduction in cellular infiltration was as follows: HA-coated DEX-CSNPs > DEX-CSNPs > DEX-AqS > LIU. The results of the present investigation as shown in Figure 8 are in accordance with previously published reports [72,82].

## 4. Conclusions

Our findings demonstrate that DEX release from HA-coated DEX-CSNPs could be sustained in vitro for 12 h, and the physicochemical characteristics (pH, clarity, refractive index, and viscosity) of the nano-formulation were suitable for topical ocular delivery, such that uncoated and HA-coated DEX-CSNPs were only mildly irritating to rabbit eyes. The Transcorneal passage of DEX from the CSNPs through the excised rabbit cornea was improved, and the ocular bioavailability of DEX from the CSNPs was higher than DEX-AqS. Importantly, coating the CSNPs’ surfaces with HA might improve cellular uptake of nanocarriers and improve corneal and conjunctival healing. In comparison to the topical administration of DEX-AqS, uncoated and HA-coated DEX-CSNPs markedly reduced signs and symptoms of LIU, inflammatory cell counts, protein concentration, and the levels of TNFα, IL-6, and MPO in AH. We believe that the DEX-mediated inhibition of apoptosis in uveal tissues is due to an increase in drug bioavailability over time (i.e., sustain release efficacy) afforded by the use of HA-coated and uncoated nanocarriers. In conclusion, our findings suggest that CSNPs have great potential for drug delivery, particularly, for the topical treatment of various inflammatory eye conditions. This nano-formulation may also be administered intravitreally, for instance, in the treatment of retinal disease; however, further investigations are warranted to understand the pharmacokinetics and safety profile of this administration.

## Figures and Tables

**Table 1:** Physical characteristics of uncoated and HA-coated drug-loaded CS-NPs before and after lyophilization without cryoprotectant. The data were presented as the mean of three readings with standard deviations (Mean ± SD, *n* = 3).

Parameters	Optimized DEX-CS-NPs before Lyophilization		Optimized DEX-CS-NPs after Lyophilization	
	Uncoated	HA-Coated	Uncoated	HA-Coated
Particle size (nm)	310.4 ± 12.4	337.3 ± 14.2	356.8 ± 14.1	368.5 ± 14.4
Polydispersity Index	0.142 ± 0.021	0.179 ± 0.078	0.248 ± 0.041	0.325 ± 0.021
Zeta potential (mV)	+31.4 ± 4.1	−5.7 ± 1.3	+32.2 ± 2.1	−6.2 ± 1.4

**Table 2:** Physical characteristics of uncoated and HA-coated DEX-loaded CS-NPs after lyophilization with mannitol (2.5%, *w*/*v*) as cryoprotectant. The data were presented as the mean of three readings with standard deviations (Mean ± SD, *n* = 3).

Formulation	Uncoated CS-NPs	HA-Coated CS-NPs
	(0.4:0.6/TPP:CS)	(0.4:0.6/TPP:CS)
Particle size (nm)	361.9 ± 14.3	379.3 ± 13.9
Polydispersity Index (PDI)	0.194 ± 0.075	0.209 ± 0.084
Zeta potential (mV)	+31.2 ± 1.9	−5.6 ± 1.2
Encapsulation (%EE)	73.6 ± 4.6	71.1 ± 3.4
Drug loading (%DL)	6.9 ± 0.4	5.5 ± 0.2
^†^ Aggregation	# #	#

**^†^** Aggregation: “#” = low (minimum), and “# #” = intermediate (medium).

**Table 3:** Physicochemical characteristics evaluated at ambient temperature of uncoated and HA-coated DEX-loaded CS-NPs after lyophilization with mannitol (2.5%, *w*/*v*) as cryoprotectant. The data were presented as the mean of three readings with standard deviations (Mean ± SD, *n* = 3).

Parameters	Time Points	Uncoated NPs	HA-Coated NPs
Clarity	Initially	Clear and transparent	Clear and transparent
	After 3 months	Clear and translucent	Clear and transparent
Refractive index	Initially	1.34 ± 0.07	1.35 ± 0.12
	After 3 months	1.35 ± 0.09	1.35 ± 0.31
pH	Initially	6.69 ± 0.34	6.81 ± 0.21
	After 3 months	7.15 ± 0.23	7.21 ± 0.03
Viscosity (mPa.s)	Initially	30.74 ± 1.49	33.76 ± 3.12
	After 3 months	34.73 ± 2.19	37.54 ± 2.09

**Table 4:** Corneal permeation parameters of DEX-containing formulations. The data were presented as the mean of three readings with standard deviations (Mean ± SD, *n* = 3).

Corneal Permeation Parameters	DEX-AqS (0.1%, *w*/*v*)	Uncoated CS-NPs	HA-Coated CS-NPs
Cumulative amount of DEX permeated (µgcm^−2^ at 0.5 h)	58.44 ± 3.04	28.36 ± 2.05	11.86 ± 3.12
Cumulative amount of DEX permeated (µgcm^−2^ in 6 h)	66.86 ± 3.51	59.52 ± 3.67	69.32 ± 4.58
pH	7.12 ± 0.08	6.69 ± 0.34	6.81 ± 0.21
Steady-state flux, *J* (µgcm^−2^h^−1^)	1.76 ± 0.13	8.27 ± 0.49	17.81 ± 0.43
Enhancement ratio	---	4.70 ± 0.39	10.14 ± 0.92
Permeability coefficient, *P* (cmh^−1^)	(3.52 ± 0.25) × 10^−3^	(16.53 ± 0.99) × 10^−3^	(35.62 ± 0.86) × 10^−3^

**Table 5:** Pharmacokinetics of dexamethasone in aqueous humor following topical application of different formulations. The data were presented as the mean of three readings with standard deviations (Mean ± SD, *n* = 3).

Pharmacokinetic	Numerical Values for		
Parameters	DEX-AqS (0.1%, *w*/*v*)	DEX-CSNPs	HA-Coated DEX-CSNPs
t_1/2_ (h)	2.18 ± 0.37	5.44 * ± 0.70	7.34 * ± 1.22
T_max_ (h)	4.00 ± 0.00	6.00 ± 0.00	6.00 ± 0.00
C_max_ (ngmL^−1^)	809.26 ± 45.51	561.79 ± 40.51	584.32 ± 50.74
AUC_0–24_ (ngmL^−1^.h)	2826.71 ± 219.84	5294.19 * ± 687.36	6691.48 * ± 570.10
AUC_0-inf_ (ngmL^−1^.h)	2830.95 ± 224.12	5727.33 * ± 897.67	7774.81 * ± 489.53
AUC_0–24/0-inf_	0.99 ± 0.001	0.93 ± 0.02	0.86 ± 0.04
AUMC_0-inf_ (ngmL^−1^.h^2^)	11,458.50 ± 1239.01	57,896.82 * ± 14,377.82	99,040.13 * ± 15,826.01
MRT_0-inf_ (h)	4.04 * ± 0.12	10.01 * ± 0.89	12.73 * ± 1.77

* *p* < 0.005 versus DEX-AqS.

## Data Availability

The data presented in this study are available on request from the corresponding author.

## Abbreviations

DEX	Dexamethasone sodium phosphate
CS	Chitosan
TPP	Tripolyphosphate sodium
NPs	Nanoparticles
CSNPs	Chitosan nanoparticles
DEX-CSNPs	DEX-loaded chitosan nanoparticles
AqS	Aqueous suspension
ZP	Zeta potential
PDI	Polydispersity index
AH	Aqueous humor
HA	Hyaluronic acid
LPS	Lipopolysaccharide
LIU	Lipopolysaccharide-induced uveitis
PMN	Polymorphonuclear
BCL2	B-cell lymphoma 2
MPO	Myeloperoxidase
NF-κB	Nuclear factor kappa B
TNFα	Tumor necrosis factor α
IL	Interleukin
COX-2	Cyclooxygenase-2
REC	Research Ethics Committee
